# Hepatoma-derived growth factor and nucleolin exist in the same ribonucleoprotein complex

**DOI:** 10.1186/1471-2091-14-2

**Published:** 2013-01-10

**Authors:** Stephanie Bremer, Katharina Klein, Angela Sedlmaier, Mekky Abouzied, Volkmar Gieselmann, Sebastian Franken

**Affiliations:** 1 Institute of Biochemistry and Molecular Biology, University of Bonn, Nussallee 11, Bonn, 53115, Germany; 2 Faculty of Pharmacy, University of El-Minia, El-Minia, Egypt; 3 Present address: Life-Science-Inkubator, Ludwig-Erhard-Allee 2, Bonn, 53175, Germany

**Keywords:** Nucleolin, Bcl-2, Ribonucleoproteins

## Abstract

**Background:**

Hepatoma-derived growth factor (HDGF) is a protein which is highly expressed in a variety of tumours. HDGF has mitogenic, angiogenic, neurotrophic and antiapoptotic activity but the molecular mechanisms by which it exerts these activities are largely unknown nor has its biological function in tumours been elucidated. Mass spectrometry was performed to analyse the HDGFStrep-tag interactome. By Pull–down-experiments using different protein and nucleic acid constructs the interaction of HDGF and nucleolin was investigated further.

**Results:**

A number of HDGFStrep-tag copurifying proteins were identified which interact with RNA or are involved in the cellular DNA repair machinery. The most abundant protein, however, copurifying with HDGF in this approach was nucleolin. Therefore we focus on the characterization of the interaction of HDGF and nucleolin in this study. We show that expression of a cytosolic variant of HDGF causes a redistribution of nucleolin into the cytoplasm. Furthermore, formation of HDGF/nucleolin complexes depends on bcl-2 mRNA. Overexpression of full length bcl-2 mRNA increases the number of HDGF/nucleolin complexes whereas expression of only the bcl-2 coding sequence abolishes interaction completely. Further examination reveals that the coding sequence of bcl-2 mRNA together with either the 5′ or 3′ UTR is sufficient for formation of HDGF/nucleolin complexes. When bcl-2 coding sequence within the full length cDNA is replaced by a sequence coding for secretory alkaline phosphatase complex formation is not enhanced.

**Conclusion:**

The results provide evidence for the existence of HDGF and nucleolin containing nucleoprotein complexes which formation depends on the presence of specific mRNAs. The nature of these RNAs and other components of the complexes should be investigated in future.

## Background

HDGF (Hepatoma-derived growth factor) is the eponymous member of a growth factor family comprising HDGF and five additional proteins (HRP1-4, LEDGF). HDGF was initially purified from serum-free supernatant of human hepatoma cell line HuH-7 [1,2]. HDGF contains a characteristic modular structure composed of an N-terminal region (amino acid residues 1–100; HATH region) conserved among all members of the family and an acidic C-terminus unique to HDGF [3,4]. Two nuclear localization signals, one in the HATH region, and in the carboxy-terminal domain are prerequisites for the mainly nuclear localization of HDGF [5,6]. However, in some cells HDGF appears also in the cytoplasm [1,7].

In several studies it has been shown that recombinant HDGF is implicated in cellular proliferation [8-11]. Furthermore HDGF has been suggested to be neurotrophic and involved in organ development and regeneration [5,7,9,11-15]. However, no obvious biochemical or morphological phenotype could be detected in a HDGF-deficient mouse model [16].

Over the last decade, HDGF research focuses on its possible role in tumour induction and/or tumour progression and metastasis. HDGF is highly expressed in a wide variety of carcinomas and has been shown to be a valid prognostic marker [17-27]. Beside its proliferative activity HDGF was reported to have angiogenic as well as antiapoptotic activity thereby making it an interesting candidate for tumour therapy [28-31]. The latter activity may be explained by a modulation of the expression of members of the bcl-2 family of pro- and antiapoptotic factors in human cancer cells by HDGF [28,32]. Besides this, the molecular mechanisms of the antiapoptotic activity of HDGF are poorly understood.

Here we describe the identification of proteins copurifying with HDGFStrep-tag on Strep-Tactin®-Macroprep beads. By transfecting a DNA construct coding for an HDGFStrep-tag fusion protein, we were able to purify HDGFStrep-tag containing protein complexes and identified 98 possible interaction partners of HDGF. Beside ribosomal proteins, heterogenous ribonucleoproteins, and members of the cellular DNA-repair machinery we identified nucleolin as a possible HDGF interacting protein by mass spectrometry and Western Blot. Similar to HDGF nucleolin was reported to play an important role in tumour biology by regulating proliferative, apoptotic and angiogenic signalling pathways [33,34]. We therefore focused in our study on a possible interaction of HDGF and nucleolin. The presented experiments reveal that the interaction of HDGFStrep-tag and nucleolin is RNA-dependent and show that HDGF is able to regulate the cellular distribution of nucleolin. Overexpression of bcl-2 mRNA enhances complex formation of nucleolin and HDGFStrep-tag in HeLa cells. Regarding our results we therefore propose a RNA-containing nucleolin-HDGF protein complex possibly regulating the cellular amount of bcl-2 mRNA.

## Methods

### Cell culture

HEK 293 and HeLa cells were grown at 37°C under 95%air/5% CO_2_ in Dulbecco’s Modified Eagle Medium (DMEM) supplemented with Glutamax®, 10% foetal calf serum, and 1 mM penicillin/streptomycin. Cells were splitted all 3 days and experiments were carried out between passages 5–15. HEK 293 cells were transfected using calcium phosphate precipitation. Briefly, 2 × 10^6^ cells were seeded on 10 cm plates the day before transfection. 65 μl of a 2 M calcium chloride solution is mixed with 10 μg of DNA in 500 μl H_2_O. This solution is dropped under stirration to 500 μl of Hepes buffered saline pH 7.13. After 30 min of incubation at room temperature the mixture is dropped to cells under mild rotation. HeLa cells were transfected with Turbofect (Fermentas) following the instruction of the manufacturer. All tissue culture reagents were obtained from Invitrogen.

### Plasmids

Sequence of murine HDGF was cloned into pCDNA3 vector (Clontech). To generate a Strep-tag® fusion protein, sequence of murine HDGF was inserted into a StreptagpCDNA3 vector with the Strep-tag® at the c-terminus. Point mutation in the nuclear localization signal 2 (NLS2) sequence (S155/170 to N155/170) of murine HDGF was generated by PCR and inserted into pCDNA3 vector.

DNAs coding for the different bcl-2 variants were generated by PCR using specific oligonucleotides given below and were inserted into pCDNA3 vector.

Bcl-2 cds sense. gcaagcttctgggaaggatggcgca

Bcl-2 cds antisense. gcgaattctgttgacttcacttgtggccc

Bcl-2 ARE1 antisense. gcgaattcgatggtgatccggccaacaac

Bcl-2 3^′^UTR antisense. gcgcggccgcccaggatgtacagataacccccat

Bcl-2 5^′^UTR sense. gcaagcttgtaagctctggagagtgctgaagat

Combinations of primers were Bcl-2 5^′^UTR sense and Bcl-2 3^′^UTR antisense (for FL Bcl-2), Bcl-2 cds sense and antisense (for cds), Bcl-2 cds sense with Bcl-2 ARE1 antisense (for ARE1), Bcl-2 cds sense with Bcl-2 3^′^UTR antisense (for 3UTR) and Bcl-2 5^′^UTR sense with Bcl-2 cds antisense (for 5UTR).

### Protein extraction

Whole cell lysates from transfected cells were prepared by incubating cell pellets (10 × 10^6^) in 250 μl ice-cold buffer L containing 50 mM Tris–HCl pH 7.4, 150 mM NaCl, 7.5% Glycerol and 1 mM EDTA supplemented with proteinase inhibitors (1 μg/ml pepstatin, 1 μg/ml leupeptin, 2 mM pefabloc) for 30 min on ice. Suspensions were sonicated three times for 10 s and centrifuged at 13.000 × g for 30 min at 4°C. Supernatants were used for analysis.

### Protein purification

Lysates from transfection experiments were either affinity-purified with Strep-Tactin®-Macroprep beads or spin columns (IBA). Briefly columns were equilibrated in 500 μl buffer W (100 mM Tris–HCl pH 8.0, 150 mM NaCl, 1 mM EDTA) and centrifuged at 700 × g for 1 min. Lysates were applied onto the columns and centrifuged two times at 700 × g for one minute. Columns were washed four times with 100 μl buffer W and centrifuged each time at 13.000 × g for 30 s. Proteins were eluted with buffer E (buffer W containing 2.5 mM Biotin) and centrifuged for one minute at 700 × g followed by thirty seconds at 13.000 × g. Beads were equilibrated with buffer L and incubated with lysates for 2 h at 4°C. Beads were washed four times with 500 μl buffer W and centrifuged for 3 min at 3000 × g. Supernatants were removed and beads were either directly digested with trypsin for mass spectrometry analysis or eluted with 30 μl buffer E. Eluate fractions were analyzed by Western blot and mass spectrometry.

For affinity purification of endogenous HDGF rabbit anti-human HDGF antibody was immobilized on NHS activated beads (1 μg/μl bead). 100 μl of these beads were filled into a spin column (PIERCE) and washed with 10 column volumes of PBS. HeLa whole cellular extracts were prepared as described above and loaded onto the HDGF affinity column. After washing the column with PBS containing 300 mM NaCl bound protein was eluted with 50 mM citric acid pH 3. A column containing rabbit IgG was used as a control. No elution of HDGF or nucleolin was detected from this column.

### Western blot analysis

Proteins were separated on a 10% SDS-PAGE gel and electrotransferred to a PROTRAN nitrocellulose transfer membrane (Whatman). Free binding sites were blocked with 5% skimmed milk in Tris–HCl buffered saline (TBS) pH 7.0 for one hour at room temperature. Primary antibodies used were monoclonal anti nucleolin antibody (H-6, Santa Cruz), peroxidase coupled Strep-Tactin® (IBA) and polyclonal anti HDGF antibody. Membranes were washed three times for 10 minutes with TBS supplemented with 0.05% Tween20 (TBS/T). For detection peroxidase coupled goat anti rabbit or goat anti mouse antibodies were used. Membranes were washed again three times with TBS/T and once with TBS before Western blotting chemiluminescent substrate (Pierce) was applied.

### Immunocytochemical staining

Briefly, cells were transfected with either mouse HDGF wild type or mouse HDGF-NLS2 mutant with Turbofect reagent (Fermentas) following the instructions of the manufacturer. After 48 hours, HeLa cells were fixed by incubation in ice-cold methanol for 10 min on ice. Coverslips were washed three times with TBS and blocked with 3% bovine serum albumin (BSA) in TBS for 30 min at room temperature. Antigens were detected by incubation with polyclonal rabbit anti HDGF antibody (1:200) and monoclonal mouse anti nucleolin antibody (1:800, Santa Cruz) in 1% BSA in TBS for one hour at room temperature. Bound antibodies were visualized using Cy3-conjugated goat anti rabbit (1:500) or Alexa 488-conjugated goat anti mouse (1:400) antibodies (Dianova). Cells were counterstained with 4′6-diamidine-2-phenylidole-dihydrochloride (DAPI). Coverslips were mounted in Mowiol and analyzed by epifluorescence microscopy on an Axiovert 100 M instrument (Zeiss, Jena, Germany).

### RNAse digestion

Cell extracts were incubated with RNAse A (Fermentas) prior to protein purification. Briefly, cell lysates were treated with 25 μg/ml RNAse A at 4°C over night under mild rotation. Protein purification was carried out as described previously.

### RT-PCR

Cells were transfected as described and 48 h after transfection RNA was isolated using 8 ml TRIzol™ (Invitrogen) according to manufacturer’s instruction. Five hundred nanogram RNA was used for cDNA synthesis using Oligo dT-primer and the ReverdAid Kit (Qiagen) as recommend by the supplier. One microliter of cDNA was used for PCR. Primer sequences for 5^′^UTR of bcl-2 were 5^′^GCAAGCTTGTAAGCTCTGGAGAGTGCTGAAGAT3^′^ (sense) and 5^′^GCGAATTCCCTTCCCAGAGGAAAAGC3^′^ (antisense) and for GAPDH were 5^′^GATGACATCAAGAAGGTGGTGA3^′^ and 5^′^CTGTAGCCAAATTCGTTGTCAT3^′^. The PCR program consisted of initial denaturation at 95°C for 30 s, annealing at 54°C for 30 s and extension at 72°C for 1 min for 25 cycles in case of the bcl-2 5^′^UTR and 20 cycles for GAPDH. The specificity of all PCR reactions was tested by parallel reactions using water instead of cDNA (not shown). The PCR products were subjected to agarose gel electrophoresis and visualized by ethidium bromide staining.

### Mass spectrometry

For analysis peptides generated by tryptic digest of Strep-Tactin®-Macroprep beads were separated using an Ultimate 3000 RSLCnano system (Dionex-LC Packings, Idstein, Germany). Samples were loaded onto a trapping column (Acclaim PepMap Nanotrap, 75 μm × 20 mm) by the loading pump of the system operating at 5 μL/min, and 0.1% trifluoroacetic acid in water was used as mobile phase. After 6 min, valve was switched and the sample was eluted onto the analytical separation column (Acclaim PepMap RSLC, 75 μm × 150 mm), using a flow rate of 300 nL/min. The mobile phases used were H_2_O/0.1% formic acid (v/v) for buffer A and 80% ACN/0.1% formic acid (v/v) for buffer B. Peptides were resolved by gradient elution using a gradient of 2 − 55% buffer B over 30 min, followed by a gradient of 50 − 90% buffer B over 2 min. After 5 min at 90% B the gradient returned to 5% buffer B preparing for the next run. Column effluent was monitored using a 3 nL UV flow cell (214 nm).

Mass spectrometric analysis was done via ESI-MS/MS using a LTQ-Orbitrap Velos mass spectrometer (Thermo Fisher, Bremen, Germany) equipped with a nano-electrospray ion source. The mass spectrometer was operated in the data dependent mode. Survey MS scans were acquired in the orbitrap with the resolution set to 30.000. Up to 15 most intense ions per scan were fragmented and analyzed in the linear ion trap.

The raw files were processed using Proteome Discoverer software version 1.3 (Thermo). The peak list files were searched against Swissprot human database by the MASCOT search engine. The initial parent and fragment ion maximum mass deviation were set to 10 ppm and 0.8 Da, respectively. The search included variable modifications of oxidation of methionine and carbamidomethylation of cysteine. The false discovery rate was set to 0.01.

Gene ontology data were added by use of the AmiGO web tool ([35]http://amigo.geneontology.org).

For peptide analysis after SDS-PAGE proteins of interest were cut from Coomassie stained gels and processed using a Trypsin Profile IGD Kit (Sigma-Aldrich, USA) following the manufacturer’s instructions. For analysis eluted peptides were separated using an Ultimate 3000 LC system (Dionex-LC Packings, Idstein, Germany). Samples were loaded onto a monolithic trapping column (PepSwift, 200 μm × 5 mm) by the loading pump of the system operating at 10 μL/min, and 0.1% Heptafluorobutyric acid in water was used as mobile phase. After 5 min, valve was switched and the sample was eluted onto the analytical separation column (PepSwift monolithic capillary column, 200 μm × 50 mm), using a flow rate of 500 nL/min. The mobile phases used were H_2_O/0.1% Formic acid (v/v) for buffer A and 100% Acetonitril (ACN)/0.1% Formic acid (v/v) for buffer B. Peptides were resolved by gradient elution using a gradient of 5 − 50% buffer B over 20 min, followed by a gradient of 50 − 90% buffer B over 1 min. After 5 min at 90% B the gradient returned to 5% buffer B preparing for the next run. Column effluent was monitored using a 3 nL UV flow cell (214 nm).

Mass spectrometric analysis was done via online ESI-MS/MS using an HCTUltra ion trap mass spectrometer (Bruker Daltonics, Bremen, Germany). All measurements were carried out in positive ion mode. MS-spectra were acquired in standard-enhanced mode between 300 to 2000 m/z at a rate of 8,100 m/z/sec. Fragmentation of peptides from MS-spectra using CID was done in Auto-MS2 mode, selecting precursor ions according to the following parameters: number of precursor ions = 5, minimal ion intensity = 10,000, ion excluded after 2 spectra, exclusion release after 1 min. MS2 data acquisition was done in ultrascan mode with a scan range of 50–3000 m/z at a scan speed of 26,000 m/z/sec.

Raw MS data for each LC run were processed using DataAnalysisTM version 4.0. The spectrum was screened for compounds using the software’s AutoMS/MS search feature applying following parameters: intensity threshold = 10,000; max number of compounds = 500; retention time = 0.4. Identified compounds were subsequently deconvoluted and exported for protein database comparison with BioToolsTM version 3.1. In BioToolsTM the exported compounds were run against an in-house SwissProt v51.6 database using the Mascot 2.2.02 algorithm. The searches were carried out using the following parameters: enzyme = trypsin; missed cleavages = 1; taxonomy = All entries; variable modifications = oxidation (M) and carbamidomethylation (C); peptide tolerance = 300 ppm; MS/MS tolerance = 1.1 Da; significance threshold p = 0.05.

## Results

### Affinity purification and identification of components of HDGF containing protein complexes

**Figure 1:**
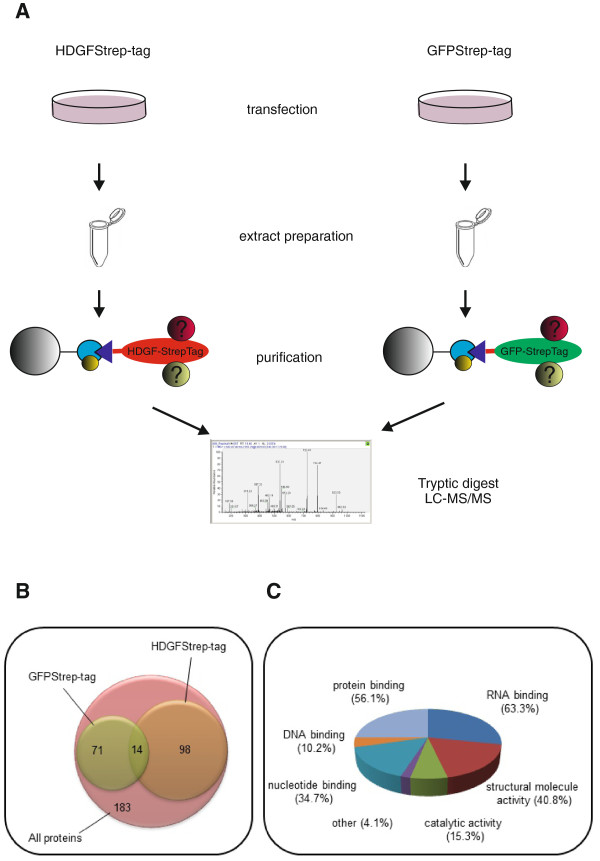
**Mass spectrometry analysis of purified HDGF protein complexes. **HDGF interaction partners were isolated using Strep-Tactin®-MacroPrep matrices as affinity resin to purify HDGFStrep-tag proteins. Trapped proteins were digested by trypsin and peptides analyzed by ESI-MS/MS. (**A**) Flow chart of the experiment. (**B**) Venn diagramm showing the overlap between HDGFStrep-tag and GFPStrep-tag interactome. (**C**) Pie chart of molecular functions of HDGFStrep-tag interaction partners (see also Additional file 4).

To learn more about how HDGF might exert its biological effects we decided to identify interaction partners of the protein after overexpression in HEK293 cells by taking advantage of the stringent purification scheme of the Strep-tag/Strep-Tactin® system [36,37]. Protein lysates from HEK cells expressing HDGFStrep-tag or as a control GFP-StrepTag fusion proteins were applied to a Strep-Tactin® MacroPrep matrix. After washing, beads were incubated with trypsin and released tryptic peptides were analysed by ESI-MS/MS (Flow chart of the experiment see Figure 1A). To control reproducibility this experiment was performed four times each for HDGF and GFP, starting with the transfection of the DNA constructs coding for the Strep-tagged proteins into HEK293 cells. Results were searched against Swissprot human protein database using Mascot search engine and all proteins identified by two or more peptides and with a sum PSM (peptide spectrum matches) of higher then nine were included into a result list. This list contained 112 and 85 interacting proteins for HDGFStrep-Tag and GFPStrep-tag lysates, respectively, of which 14 were identified in both samples (Figure 1B; complete lists in Additional files 1, 2 and 3). Subtraction of the overlapping proteins resulted in a list of 98 proteins only found in the HDGFStrep-Tag interactome. GO molecular function analysis of these proteins shows that many of them have binding functions (84.7%), including protein binding (56.1%) and nucleic acid binding, especially RNA binding (63.3%) (Figure 1C and Additional file 4). Besides HDGF, the highest score and the highest number of PSMs were obtained for nucleolin (Additional file 1), a protein known to be involved in proliferation, apoptosis and neoangiogenesis similar to what was reported for HDGF. Due to this surprising overlap with HDGF function we focused on nucleolin in our further analysis.

**Figure 2:**
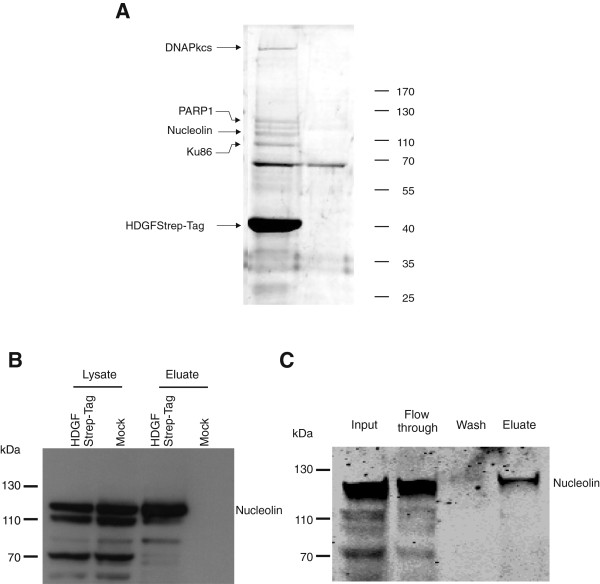
**Verification of nucleolin copurification with HDGF. **(**A**) Analysis of most abundant HDGFStrep-tag copurifying proteins by Coomassie stained SDS-PAGE. Proteins purified via Strep-Tactin®-MacroPrep matrices after transfection of cells with HDGFStrep-tag or untagged HDGF (Mock) were separated using SDS-PAGE and visualized by Coomassie staining. Visible proteins specific for HDGFStrep-tag sample correspond to Ku86 (86 kDa), nucleolin (110 kDa), PARP-1 (113 kDa) and the catalytic subunit of DNA-PK (460 kDa). (**B**) To confirm mass spectrometric results, lysates of HEK293 cells expressing HDGFStrep-tag fusion proteins and their eluate fractions from HDGFStrep-tag purification were examined by Western blot with a specific nucleolin antibody. Nucleolin is only present in eluates from cells expressing HDGFStrep-tag fusion proteins. (**C**) To investigate whether endogenous HDGF also interacts with nucleolin a cellular extract of untransfected HeLa cells was loaded onto a human HDGF antibody column. Non-specific proteins were washed out with 300 mM NaCl. Specifically binding proteins were eluted with 50 mM citric acid pH 3. Western blot analysis with a monoclonal anti nucleolin antibody confirmed copurification of nucleolin with endogenous HDGF.

First of all we confirmed copurification of nucleolin with HDGF by an independent SDS-PAGE gel based identification strategy. Therefore, HDGFStrep-tag copurifying proteins where not digested directly on the beads but were eluted with biotin, separated by SDS-PAGE, and the most abundant coeluting proteins were stained by Coomassie Brilliant Blue (Figure 2A). Besides a strong signal corresponding to HDGFStrep-tag additional proteins of higher molecular weight could be detected specifically copurifying in the presence of HDGFStrep-tag but not in the eluate prepared from cells transfected with a control construct. Peptides released from gel pieces by tryptic digest were analysed by nanoLC-ESI-Iontrap mass spectrometry. MASCOT database searches of the peptide sequences revealed highly significant protein identifications for nucleolin, Ku86, PARP1 and DNA-Pkcs. In detail, thirteen different peptides for nucleolin resulting in a sequence coverage of 22%, fourteen peptides for Ku86 (26% sequence coverage), fifteen peptides for PARP1 (18% sequence coverage) and twenty peptides for DNA-Pkcs (8% sequence coverage) were identified (see Additional file 5). To confirm our mass spectrometry results we performed Western blotting on the HDGFStrep-tag eluate fractions using a specific nucleolin antibody. Results in Figure 2B demonstrate the specific coelution of nucleolin from the HDGFStrep-tag resin using transfected HEK293 cells. In addition copurification of Ku86 could also be proofed by Western blotting (Additional file 6).

### HDGF is part of a RNA-dependent nucleolin containing complex and influences cellular distribution of nucleolin

Influence of nucleolin on cellular mRNA content in HeLa cells has been studied extensively in a recent study [38]. Therefore, extracts from untransfected HeLa cells were passed over a human HDGF antibody column to investigate whether the nucleolin-HDGF complex formation can also be observed with endogenous HDGF. Unspecifically bound proteins were removed by washing with high salt containing buffer. Proteins bound to the column via the HDGF antibody were eluted with 50 mM citric acid pH 3. Eluted proteins were separated by SDS-PAGE, transferred onto nitrocellulose membrane and subjected to Western blot analysis. Immunoblotting with a monoclonal nucleolin antibody confirmed specific binding of nucleolin to the HDGF antibody column (Figure 2C). Thus, the interaction of HDGF with nucleolin is not a specific feature of the HDGFStrep-tag fusion proteins but occurs also with unfused endogeneous protein. Interestingly, for both strategies described here only the full length nucleolin showed efficient copurification together with HDGF whereas smaller forms detected by the specific antibody against nucleolin did not purified together with HDGFStrep-tag as well as endogenous HDGF. This observation points to the specificity of the purification protocol.

**Figure 3:**
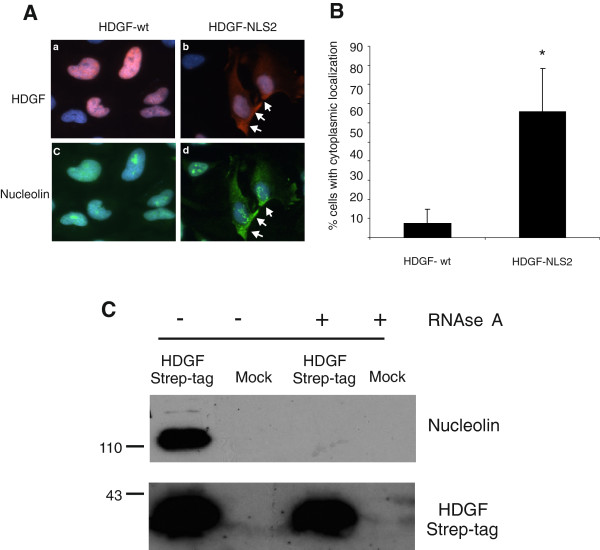
**HDGF changes intracellular distribution of nucleolin. **(**A**) To examine HDGF/nucleolin complex formation in HeLa cells, cells were transfected with either wild type HDGF or a HDGF-NLS2 mutant expression vector and fixed after 48 hours with methanol. Cells transfected with wild type HDGF (HDGFwt) show predominant nuclear localization of HDGF (red, Figure 3B panel a) as well as nucleolin (green, Figure 3B panel c). In contrast, induction of cytoplasmic expression of HDGF-NLS2 leads to a significant increase of cytoplasmic HDGF (red, Figure 3B panel b) as well as nucleolin (green, Figure 3B panel d). Cells were counterstained with DAPI (blue). (**B**) HDGF positive cells were counted (n = 100) and localisation of nucleolin was analysed. 67% of HDGF-NLS2 transfected cells show cytoplasmic localisation of nucleolin in comparison to only 7% of wild type transfected cells. *p < 0.001. (**C**) Protein lysates from HDGFStrep-tag expressing HeLa cells were incubated with or without RNAse A (25 μg/ml) over night at 4°C followed by affinity purification via Strep-Tactin®-MacroPrep spin columns. Proteins from eluate fractions were subjected to Western blot analysis using a specific nucleolin antibody (upper panel) or with peroxidase coupled Strep-Tactin® to detect HDGFStrep-tag (lower panel). In samples treated with RNAse A a complete loss of interaction can be observed.

HDGF is predominantly a nuclear protein with low expression in the cytoplasm. Recent data, however, suggest a cytoplasmic localization in certain tumours. Nucleolin is primarily a nucleolar protein with the ability to shuttle between nucleus, cytoplasm and cell surface. To investigate whether cellular localization of nucleolin can be manipulated by HDGF, HeLa cells were transfected with an expression vector encoding for a murine HDGF cDNA with a mutation in the NLS2 (Nuclear Localization Signal 2) sequence which has been shown before to cause a predominant cytoplasmic localization of HDGF (HDGF-NLS2). Cells transfected with the HDGF-NLS2 showed strong nucleolin expression in the cytoplasm (Figure 3A, panel d) whereas in cells with wild type HDGF nucleolin was retained mainly in the nucleus (Figure 3A, panel c). To quantify this effect, we counted cells (n = 100) positive for HDGF staining and analysed these cells with regard to their localisation of nucleolin. We distinguished nuclear and cytoplasmic localization. 67% of cells transfected with HDGF-NLS2 showed translocation of nucleolin from nucleus to cytoplasm. In comparison, only 7% of wild type HDGF transfected cells showed this effect (Figure 3B). Therefore, redistribution of HDGF into the cytoplasm leads to substantial cytoplasmic nucleolin localization. This observation is strong evidence of functionally relevant in vivo interaction of HDGF and nucleolin.

HDGF was shown to exhibit binding activity upon ribosomal RNAs [39]. In accordance to this we observed copurification of RNA from HeLa as well as HEK 293 cells during purification of HDGFStrep-tag. Together with 2 μg of protein an average of 15 μg RNA could be eluted from HDGFStrep-tag MacroPrep beads. In comparison unspecific RNA binding of beads not loaded with protein was less than 1 μg (data not shown). Nucleolin was reported to interact with other proteins in a RNA-dependent manner [40]. Therefore we investigated whether HDGFStrep-tag-nucleolin interaction depends on the presence of RNA. Therefore, cell lysates of HeLa cells expressing HDGFStrep-tag fusion proteins were incubated with 25 μg/ml RNAse A prior to the affinity purification of HDGFStrep-tag containing protein complexes. This treatment completely abolished the interaction of HDGFStrep-tag and nucleolin as detected by Western blotting against nucleolin (Figure 3C).

### HDGF-nucleolin complex formation is mediated by bcl-2 full length mRNA

To investigate whether HDGF-nucleolin copurification occurs because of HDGFs general RNA binding activity or whether it depends on the presence of certain mRNAs we screened the literature for functional overlaps in HDGF and nucleolin activity. The latter is known to regulate cellular bcl-2 protein amount by binding to and stabilizing its mRNA. Interestingly, HDGF was also reported to manipulate cellular bcl-2 content [28,40].

**Figure 4:**
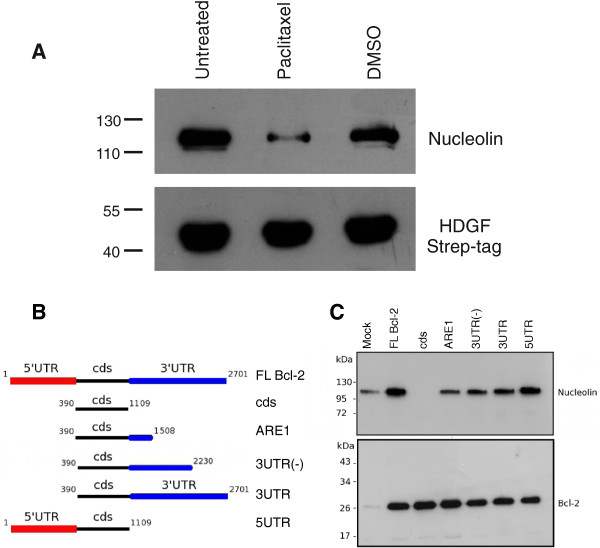
**HDGF and nucleolin complex formation depends on bcl-2 full length mRNA. **(**A**) To investigate a possible involvement of bcl-2 mRNA in HDGF-nucleolin complex formation cells were treated with Paclitaxel and protein lysates were examined for nucleolin copurification as described above. Incubation with the bcl-2 mRNA destabilizing agent Paclitaxel led to a significant decrease of nucleolin copurification. (**B** + **C**) Different deletion constructs of bcl-2 mRNA were generated and cotransfected together with HDGFStrep-tag to investigate their influence on the copurification of nucleolin. The full-length bcl-2 mRNA (FL Bcl-2) had a positive whereas the coding sequence of bcl-2 (cds) had a negative effect on the amount of coprecipitated nucleolin. Interestingly, addition of 400 bp of the 3^′^UTR (ARE1) containing a known nucleolin binding motive was already sufficient to rescue the negative effect on the amount of precipitated nucleolin. Furthermore, beside the 3′UTR also the 5^′^UTR had a rescuing effect on the interaction.

To examine whether bcl-2 mRNA might be involved in the observed complex formation we took advantage of Paclitaxel, a substance which was reported to destabilize bcl-2 mRNA [41]. If the HDGFStrep-tag-nucleolin complex depends on the presence of bcl-2 mRNA Paclitaxel treatment should reduce the amount of nucleolin copurified with HDGFStrep-tag fusion protein. Indeed, addition of 500 μM Paclitaxel to the cell culture medium of HeLa cells led to a significant decrease of nucleolin in the HDGFStrep-tag eluate fractions (Figure 4A).

To proof the role of bcl-2 mRNA in HDGFStrep-tag-nucleolin complex formation HeLa cells were transfected with a vector encoding different parts of bcl-2 mRNA and the HDGFStrep-tag fusion protein (Figure 4B). All constructs led to a similar degree of bcl-2 protein expression in transfected cells (Figure 4C lower panel). After purification of the HDGFStrep-tag fusion protein via affinity chromatography nucleolin was detected in the eluate fractions. Figure 4C shows that expression of a full length version of bcl-2 mRNA (FL Bcl-2) leads to an increased amount of nucleolin in the eluate fractions. This indicates that bcl-2- mRNA causes an enhanced interaction between HDGFStrep-tag fusion proteins and nucleolin. Nucleolin was reported to bind to the UTR’s of bcl-2 mRNA [40]. If the interaction of HDGFStrep-tag and nucleolin depends on nucleolin binding to bcl-2 mRNA one would expect that the coding sequence of bcl-2 alone will not increase the amount of nucleolin copurifying with HDGFStrep-tag. Surprisingly, transfection of the corresponding construct (cds) not only failed increasing nucleolin copurification but inhibited the complex formation of HDGF and nucleolin completely (Figure 4C). To localize bcl-2-mRNA regions responsible for HDGFStrep-tag nucleolin interaction in more detail we also transfected several vectors expressing different sections of the bcl-2-mRNA (Figure 4B). In contrast to the cds alone constructs containing one or both UTRs were able to restore the activity of the bcl-2 mRNA to increase HDGFStrep-tag nucleolin interaction. Even addition of the ARE1 that was reported to be the minimal motive for nucleolin binding to the bcl-2 mRNA was sufficient to restore the observed activity of bcl-2 mRNA.

**Figure 5:**
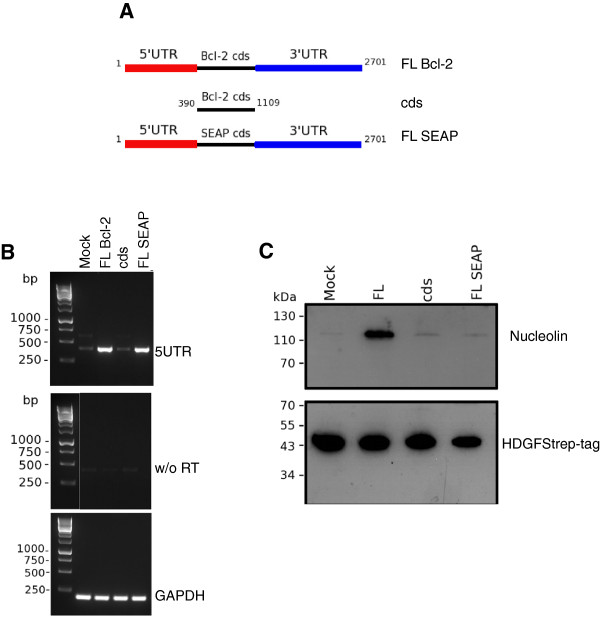
**Cds of bcl-2 mRNA is necessary for HDGF and nucleolin complex formation. **(**A**) To investigate a possible role of the coding sequence of bcl-2 (cds) it was replaced by the cds of secreted alkaline phosphatase (SEAP). (**B**) Both constructs led to a significant increase in bcl-2 UTRs in the cells as detected by RT-PCR. (**C**) Replacement of the cds of bcl-2 against the cds of SEAP was not able to increase the amount of coprecipitated nucleolin.

To examine whether the UTRs are not only necessary but sufficient for this effect, we exchanged the cds of bcl-2 in its full length construct (FL Bcl-2) for the cds of the secreted alkaline phosphatase (FL SEAP; Figure 5A). RT-PCR demonstrates the increased amount of the bcl-2 UTRs after transfection of constructs coding for the bcl-2 full length mRNA as well as the bcl-2/SEAP chimera in contrast to the cds alone (Figure 5B). Interestingly, only the full length bcl-2 construct was able to increase copurification of nucleolin demonstrating an important role of the bcl-2 cds in HDGFStrep-tag nucleolin complex formation in addition to its UTRs (Figure 5C).

## Discussion

In multiple studies it has been shown that elevated levels of HDGF in different types of tumours correlate with metastatic potential, increased malignancy and thus, a unfavourable prognosis for patients [17-22,24,25,42]. Approaches to explain these observations showed that HDGF modulates a wide variety of cellular functions ranging from proliferation to angiogenesis and cellular survival [28,29,31,32,43,44]. Regarding the latter, HDGF has been reported to exert antiapoptotic activity via the regulation of members of the bcl-2 protein family. Depending on different cell types upregulation of bcl-2 (antiapoptotic) or downregulation of bad (proapoptotic) was reported. However, whether HDGF achieves these modulations via transcriptional control mechanisms like reported for the SET and MYND domain containing 1 gene [45] or on a posttranscriptional level is completely unknown.

In this report, we investigated the HDGF interactom. Most of the proteins identified here have functions in RNA biogenesis and processing. Furthermore, we identified members of DNA repair pathways XRCC6/Ku70, XRCC5/Ku86, PARP1 and DNA-PKcs among the most prominent HDGF interaction partners. PARP-1 was shown before to form a complex with both DNA-PKcs and Ku70/Ku86 [46,47]. This complex plays an important role in non-homologous end joining a special form of DNA repair [48]. Lepourcelet and coworkers reported the correlation of HDGF reexpression with mismatch repair proficient colon carcinoma in contrast to carcinomas deficient for this type of DNA repair suggesting an involvement of HDGF in this kind of biological process [49]. Furthermore, a more global approach on HDGF interactions also found different members of DNA repair pathways as possible interaction partners of HDGF [39]. The biological relevance of this finding is subject of further investigation but the identification of all three proteins underlines the specificity of our approach.

Besides the members of DNA repair pathways nucleolin copurified with HDGF. Multiple functions have been assigned to nucleolin a RNA-binding phosphoprotein of the nucleolus. Besides its function in ribosomal biogenesis nucleolin is associated with many processes dysfunctional in tumour cells [34,50]. These properties are in line with probable HDGF functions. Most interestingly, nucleolin has been shown to be a key regulator of bcl-2 via binding and stabilizing its mRNA [40]. Therefore, to investigate possible biological relevance of our results we focused on the HDGF-nucleolin interaction. First of all using an antibody based affinity chromatography approach we demonstrated the existence of an HDGF-nucleolin containing complex also for endogenous HDGF. This is important because all other proteomic studies to date only reported interactions after overexpression of HDGF.

Nucleolin is a protein with predominant localization in the nucleus but is also able to translocate to the cytoplasm similar to what was reported for HDGF. Of interest, as shown in Figure 3, HDGF is able to regulate the cellular localisation of nucleolin in HeLa cells. This finding again underlines the in vivo relevance of the HDGF-nucleolin interaction. Previous data suggest that the phosphorylation state of nucleolin is important for the efficiency of nucleolin shuttling [51]. Whether phosphorylation/dephosphorylation signalling or other mechanisms are involved in HDGF-nucleolin shuttling has to be revealed in further studies.

Nucleolin has RNA binding activity and many interactions of nucleolin occur in the presence of RNA. Binding to ribosomal RNAs has also been recently demonstrated for HDGF [39]. Not surprisingly the observed copurification of nucleolin with HDGFStrep-tag is also dependent on RNA. Here we show that specifically bcl-2 mRNA is able to mediate HDGF-nucleolin complex formation. Regulation of bcl-2 on mRNA as well as protein level has attracted increasing attention over the last years and is frequently an obstacle on cancer treatment [52-54]. Bcl-2 mRNA stabilization and regulation of translation efficiency is regulated by nucleolin via binding to AU-rich elements (ARE) in the 3^′^untranslated region (3^′^UTR) [55,56]. Consistent with this we show that the presence of these regions is a prerequisite for the bcl-2 mRNA induced HDGF-nucleolin complex formation.

Formation of the HDGF-nucleolin complex depends on the presence of either the 3^′^ or 5^′^ UTR in combination with the coding sequence, respectively. In contrast, the coding sequence alone or its replacement by an unrelated coding sequence does not propagate complex formation. This clearly shows that the combination of UTRs and bcl-2 coding sequence is essential for HDGF-nucleolin interaction. HDGF has been shown to bind RNA rather unspecifically [39]. A model compatible with the bcl-2 mRNA dependent formation of the HDGF/nucleolin complex would be that HDGF binds the coding sequence rather unspecifically and that nucleolin is incorporated via regions of the 3 and 5^′^ UTR.

Besides its already known function as a transcriptional repressor the results described here point to a possible role of HDGF in RNA transport, stability and/or translational control. Furthermore nucleolin was shown to regulate transport of mRNA as a member of ribonucleoprotein complexes [57] and translation for example of p53 together with ribosomal protein L26 [58]. Future approaches should therefore target possible roles of HDGF in RNA transport and translational control.

It is also not known whether there is a direct interaction between HDGF and nucleolin. This finding would be interesting regarding a possible interaction of HDGF and nucleolin at the cell surface. Nucleolin was reported to function as cell surface receptor for molecules like midkine or pleiotrophin [59-61]. Interestingly, as reported for HDGF nucleolin can also bind to certain glycosaminoglycans [3,8,42,62,63] and heparin binding motifs of potential ligands can function as binding motifs for nucleolin [64]. Similar to ribonucleic acids GAGs have been demonstrated to support a wide variety of protein-protein interactions. Nucleolin therefore could possibly serve as the up to now unknown cell surface receptor for HDGF in a GAG-dependent way explaining at least some of its proliferative as well angiogenic properties as an extracellular factor during tumour formation.

## Conclusions

HDGF has been reported to be overexpressed in a wide variety of tumours and its expression correlates with an unfavourable prognosis for cancer patients. But how HDGF exactly contributes to tumourigenesis is not known up to now. Interestingly, in this study we demonstrate that nucleolin, a protein known to be involved in a set of processes relevant during tumourigenesis, can participate in these complexes and that the mRNA of bcl-2, a known regulator of apoptosis, contributes to their composition. The results presented here give evidence that beside its activity as a transcriptional repressor intracellular HDGF might exerts some of its cellular functions by posttranscriptional regulation of RNAs as a member of nucleoprotein complexes. These findings therefore contribute to the understanding of a possible role of HDGF during tumour onset and progression and are the base for a new set of biochemical and cell biological experiments addressing HDGF biology.

## Competing interests

The authors declare that they have no competing interests.

## Authors’ contribution

SB carried out the experiments and drafted the manuscript. KK and MA participated in the interactome analysis of HDGF. AS carried out interaction analysis. VG participated in study design and drafted the manuscript. SF supervised the study, took part in sample analysis and study design and drafted the manuscript. All authors read and approved the final manuscript.

## Supplementary Material

Additional file 1 **HDGF interactome. **Four biological replicates each were analyzed for HDGFStrep-tag or GFPStrep-tag copurifying proteins by ESI-MS/MS. Proteome discoverer software were used to produce multiconsensus lists of the four HDGF and the four GFP replicates. Lists were filtered for proteins with at least 2 peptide hits and at least 10 peptide spectrum matches (PSM). Afterwards proteins present in both lists were subtracted to receive the HDGF interactome. Click here for file

Additional file 2 **Multiconsensus list of four HDGF measurements. **Four biological replicates each were analyzed for HDGFStrep-tag copurifying proteins by ESI-MS/MS. Proteome discoverer software were used to produce a multiconsensus list of the four HDGF replicates. This list was filtered for proteins with at least 2 peptide hits and at least 10 peptide spectrum matches (PSM). Click here for file

Additional file 3 **Multiconsensus list of four GFP measurements. **Four biological replicates each were analyzed for GFPStrep-tag copurifying proteins by ESI-MS/MS. Proteome discoverer software were used to produce a multiconsensus list of the four GFP replicates. This list was filtered for proteins with at least 2 peptide hits and at least 10 peptide spectrum matches (PSM). Click here for file

Additional file 4 **Gene ontology annotations of the HDGF interactome. **Proteins present in the HDGF interactome (see Additional file 1) were analyzed for Biological Process, Cellular Compartment and Molecular Function via the AmiGo online analysis tool (http://amigo.geneontology.org). Click here for file

Additional file 5 **Peptide hits of proteins processed by SDS-PAGE and ESI-MS/MS. **ESI-MS/MS files were searched against Swissprot database by the MASCOT search engine. Peptide residues of the identified proteins were listed together with measured and calculated peptide masses. Click here for file

Additional file 6 **Western blot against Ku86/XRCC5. **To confirm mass spectrometric results, lysates of HEK293 cells expressing HDGFStrep-tag fusion proteins and their eluate fractions from HDGFStrep-tag purification were examined by Western blot with a specific Ku86 antibody. Ku86 is only present in eluates from cells expressing HDGFStrep-tag fusion proteins. Click here for file
