# Supplementary material for: Hepatoma-derived growth factor and nucleolin exist in the same ribonucleoprotein complex
Source: BMC Biochem. 2013 Jan 10;14:2. doi: 10.1186/1471-2091-14-2 (PMC3551658; doi:10.1186/1471-2091-14-2)
Supplement: Additional file 6 — Western blot against Ku86/XRCC5. To confirm mass spectrometric results, lysates of HEK293 cells expressing HDGFStrep-tag fusion proteins and their eluate fractions from HDGFStrep-tag purification were examined by Western blot with a specific Ku86 antibody. Ku86 is only present in eluates from cells expressing HDGFStrep-tag fusion proteins. [file 1471-2091-14-2-S6.pdf]

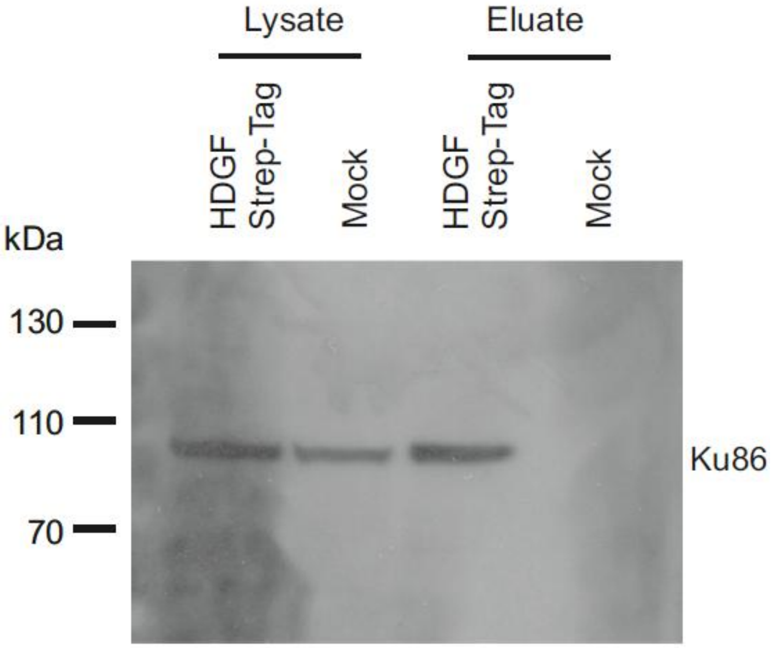

Western blot against Ku86/XRCC5

To confirm mass spectrometric results, lysates of HEK293 cells expressing HDGFStrep-tag fusion proteins and their eluate fractions from HDGFStrep-tag purification were examined by Western blot with a specific Ku86 antibody. Ku86 is only present in eluates from cells expressing HDGFStrep-tag fusion proteins.
